# Changing mortality amongst hospitalised children with Severe Acute Malnutrition in KwaZulu-Natal, South Africa, 2009 – 2018

**DOI:** 10.1186/s40795-022-00559-y

**Published:** 2022-07-12

**Authors:** S Ndlovu, C David-Govender, P Tinarwo, KL Naidoo

**Affiliations:** 1 Department of Paediatrics and Child Health, School of Clinical Medicine, College of Health Sciences, University of KwaZulu-Natal, Durban, South Africa; 2 Department of Dietetics, KwaZulu-Natal Department of Health, King Edward VIII Hospital, Durban, South Africa; 3 Department of Biostatistics, Nelson R Mandela School of Medicine, University of KwaZulu-Natal, Durban, South Africa; 4 Department of Paediatrics, King Edward VIII Hospital, Private Bag X7 Congella, Durban, 4013 South Africa

**Keywords:** Paediatrics, Severe acute malnutrition, HIV, Under-5 mortality

## Abstract

**Background:**

The under-five mortality rates of children in South Africa (SA) remain high despite successful HIV prevention and treatment programs. The in-hospital mortality of children with severe acute malnutrition remains a key obstacle. This study identifies and describes changes in the mortality of under-five children with severe acute malnutrition (SAM) following the implementation of HIV and malnutrition prevention and treatment programmes.

**Methods:**

This was a retrospective review of in-hospital mortality records and databases. The study was based at a large referral hospital in KwaZulu-Natal (KZN), where HIV and malnutrition rates are high, and SAM children are managed with standard WHO guidelines. Records of children under five years old who died from 2009 to 2018 were analysed.

**Results:**

Of the 698 under-five children who died in this period, 285 (40, 8% of all under-5 deaths) were classified as having SAM. The number of HIV-infected SAM deaths dropped significantly, especially those below six months of age, mirroring the expansion of HIV treatment and prevention programmes. Despite this and a significant drop in the proportion of SAM admissions identified, there was no change in SAM case fatality rates over the ten years. Septicaemia remained the most common cause of death in children with SAM.

**Conclusions:**

Despite significant decreases in HIV-related malnutrition deaths over ten years, the lack of change in SAM case fatality rates is a concern at this referral hospital. Standardised WHO inpatient management protocols, may require review, especially where underlying medical conditions may contribute to SAM deaths in HIV-negative children.

## Introduction

Globally, several interventions, including health system strengthening, improvements in maternal education and family income, and the establishment of the Sustainable Development Goals, have contributed to a reduction in under-five child mortality [1]. While improvements were noted in decreasing under-five mortality in South Africa (SA), these were insufficient to achieve set goals. The current under-five mortality rate remains higher than the goal to reduce it to 25 per 1000 live births by 2030 [2, 3]. This is especially true in children who have severe acute malnutrition (SAM), as defined by the World Health Organization (WHO) [4–6].

Health system strengthening in SA includes prioritising resources in managing the major causes of mortality in children, viz. HIV, tuberculosis, acute gastroenteritis, pneumonia, and SAM. Priority programmes focusing on these major drivers of under-five mortality are implemented at scale across districts and provinces and emphasise a primary health care approach [3]. With more than 40% of the population living below the poverty line, these interventions include nutrition-related programmes aimed at community prevention and institutional care of children with SAM [7]. Key nutrition programmes include nutritional support for underweight pregnant women, infant and young child feeding, growth monitoring and promotion, social protection and food provisioning, the national school nutrition programme, vitamin A supplementation and fortification of food staples with micronutrients [8–12]. In addition, the endorsement promotion, protection and support for breastfeeding with the signing of the Tshwane declaration in 2011 has influenced feeding practices, especially at the institutional level, through the implementation of the Mother-Baby Friendly Initiative [13].

Mortality review data collected through the Child Healthcare Problem Identification Programme (CHIP) showed that approximately 30% of children who die in South African hospitals have severe acute malnutrition, including larger referral hospitals. [14]. Analyses of mortality in children following discharge have advocated that a risk-based approach to inpatient management, related to malnutrition, should be a major intervention across Africa and Asia. [15]Specific WHO sanctioned programmes aimed at improving the management of hospitalised malnourished children have been introduced at scale nationally and provincially in SA since 2006 [16]. In January 2014, guidelines for the integrated management of acute malnutrition (IMAM) were introduced to monitor and implement standardised WHO protocols at the hospital level [17]. Hospital-based record-keeping with formal death reviews of children with SAM was also introduced. In June 2015 and again in March 2018, KZN provided province-specific updates on these IMAM guidelines [17]. Evaluating the impact of these programmes has been primarily based on national and provincial assessments, with gaps noted in the ability to estimate the effectiveness of overall treatment approaches for malnutrition [18–20]. This study defines referral hospitals as those having specialist paediatric services that manage children referred from facilities with no specialised care (primary healthcare facilities). Referred children include those with SAM who fail to improve or require higher care levels. According to WHO guidelines, children with SAM in these referral hospitals are also managed using the same standardised malnutrition protocols utilised in primary healthcare facilities with little or no deviation. [16]. One gap in determining the impact of nutritional programmes is assessing any changes in mortality in these referral hospitals that manage SAM children who often have complex problems.

The national scale-up of programmes to prevent mother-to-child transmission and treatment of HIV was initially introduced in 2004 with increasingly efficacious antiretroviral regimen updates until 2017 [21]. These programmes reduced the mother-to-child transmission of HIV rate dramatically [3]. However, over the same period, there appears to have been less progress in the coverage of interventions for malnutrition, pneumonia and diarrheal disease, which are now the leading causes of under-five mortality outside the neonatal period in SA [3]. In 2015/2016, KwaZulu-Natal was among the top four provinces in SA, with the highest number of under-five deaths [3, 7]. In Southern and Eastern Africa, including KZN with its very high HIV rates, much of this excess mortality has been attributed to concurrent HIV infection [22]. The interactive effects of HIV infection on increased mortality risk in malnourished children have led to calls for more information and revised international treatment guidelines in such cases [23].

Understanding the impact and effectiveness of multiple interventions to decrease SAM is critical to demographers and public health experts. In addition, it provides a basis for planning national health strategies and tracking progress toward child survival goals. This study determined changes in the mortality of children under five years of age hospitalised with SAM at a referral institution in Durban, KwaZulu-Natal (KZN), over ten years. An additional objective of the study was to determine the relationship between HIV infection and mortality among these hospitalised SAM children following the rollout of both HIV and nutrition treatment and prevention programmes.

## Methodology

This study was a retrospective cohort study reviewing in-hospital mortality records, admission and death registers, and various databases. The study was conducted in King Edward VIII Hospital (KEH) in Durban, KZN, in SA. KEH is a referral hospital that drains a large population base of urban and semi urban communities where an estimated 4% of all children live with HIV and 21, 6% exposed at birth to HIV. [24, 25]Children from these areas are referred from primary healthcare facilities to KEH with a 75 paediatric medical bed capacity with high care facilities.

The study population focused on hospitalised children with SAM who were demised in the paediatric medical wards of KEH from 01 January 2009 to 31 December 2018. This specific ten-year period was selected to determine changes following the wide-scale implementation of HIV treatment and prevention programmes during this period [21]. Second, this same period saw the uniform implementation of WHO standard guidelines for the inpatient management of SAM [17]. The study excluded a review of deaths of neonates (less than 28 days) and focused on all infants and children from 1–59 months at the time of admission.

Ethics approval from the University of Kwa Zulu–Natal (UKZN) Biomedical Research Ethics Committee (BREC/00001341/2020) and gatekeeper permission from the KwaZulu–Natal Department of Health and King Edward VIII Hospital were obtained before data collection and verification.

To obtain the data for this study, which included annual tallies and individual mortality information, the primary investigator reviewed four independent databases for the study period. All four databases were used to corroborate information and ensure minimal missing data. The King Edward Hospital Paediatric database was the primary database (KPDD). A designated specialist paediatrician working in KEH was responsible for verifying and entering all daily admissions tallies and death information from the original case records. The information in this database is also verified monthly and annually by other paediatricians in the department. For this study, repeat admissions within seven days of the previous admission were reflected as a single hospitalisation. The second database used was the district health information system (DHIS). In this provincially mandated system, nurses and clinicians verify admission and mortality data from a ward-based admission and deaths daily register (ADD triplet). The third database used was the Child Healthcare Problem Identification Programme (Child PIP). Paediatric departments across many SA hospitals utilise this database to record and systematically review child deaths, emphasising assessing modifiable factors related to these deaths. The fourth database used was that specific to children with SAM, maintained by dietitians attending to hospitalised children. Dietitians attending to malnourished children independently verified the hospitalised children’s weights, lengths, and nutritional classification.

Data for this study were obtained initially from the KPDD database and then cross-referenced with all other databases to verify recorded age, weights, lengths, nutritional classification, HIV exposure and infection status, final diagnosis, and the primary cause of death. The primary investigator then reviewed all databases for each child and entered the data into a predesigned Excel spreadsheet. Where data were conflicting, a consensus was reached following the evaluation of the original case records by two investigators (SN and KLN).

The WHO nutritional classification system, which recorded lengths as part of this classification system, recorded all children’s nutritional status. In this classification system, children were classified as severe acute malnutrition (SAM), moderate acute malnutrition (MAM), not acutely malnourished but considered at risk (NAM@risk), not acutely malnourished (NAM) or obese as defined by the WHO growth standards [4]. The definition was based on oedema or weight-for-length z score as reported by the admitting clinicians and verified by the discharging clinician and dietician.MUAC scores were not used as this documentation of this was inconsistent in the reviewed charts In children in whom the Wellcome classification system was used, where the terms kwashiorkor, marasmus or marasmic kwashiorkor were utilised, cases were reclassified according to the WHO classification system using available weights, lengths and clinical information [26]. The primary focus of this study was to analyse children classified as having SAM, as these children are generally deemed most at risk for mortality.

HIV results were obtained as documented in the mortality databases. For the study period it was routine practice to test all hospitalised children for HIV with a DNA PCR test (for those less than 18 months) and a HIV ELISA (above 18 months). For this study HIV results were recorded as HIV positive, HIV negative or HIV unknown.

The final diagnosis and primary cause of death recorded in the various databases was determined as per the treating clinician’s opinion. The diagnostic classification system used included the categories acute Gastroenteritis, (AGE), lower Respiratory Tract Infections (LRTI), confirmed /Suspected septicaemia, meningitis, cardiac failure, neurological causes, liver failure /disease, renal Failure /disease and other causes.

### Statistical data analysis

The statistical data analysis was conducted in R Statistical computing software of the R Core Team, 2020, version 3.6.3. The results are presented in the form of descriptive and inferential statistics. The descriptive statistics of numerical measurements were summarised as the minimum, maximum, quartiles, interquartile range, means, standard deviation and the coefficient of variation. Categorical variables were described as counts and percentage frequencies, where multiple bar charts were used to display these variables visually. In some instances, MS Excel was used to aid with the visuals of the trend analysis The Mann–Kendall statistical test was used to assess for trend. Depending on the distribution of the numerical variables between two independent groups, mean or median differences were assessed using either the t-test or Wilcoxon test, respectively. To determine the association between categorical variables, a chi-square test was used. When the distribution of the cross-tabulations contained an expected value of less than five, Fisher’s exact test was applied. All inferential statistical analysis tests were conducted at the 5% significance level.

## Results

**Fig. 1:**
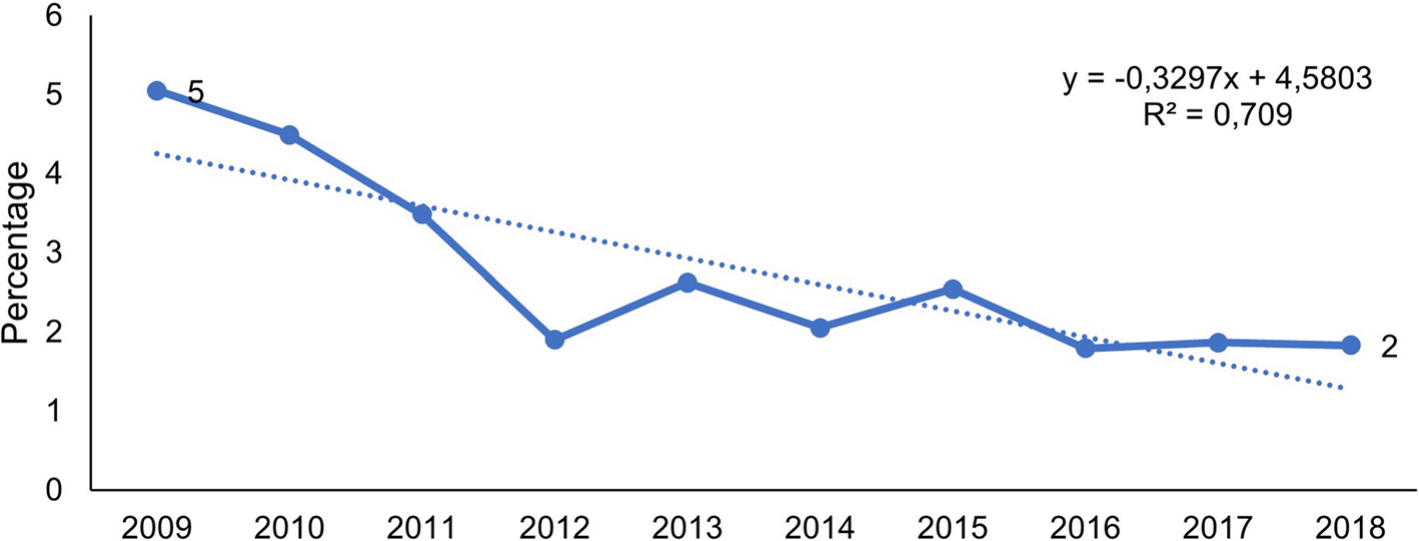
Change in percentage under-five deaths from 2009–2018

Over the ten-year study period, 698 (2, 7%) of the 25 595 hospitalised children under five years old died in King Edward VIII Hospital. During this period, the total number of under-five hospitalisations remained relatively constant between 2507 (lowest) and 2724 (highest) each year. Despite this, the percentage of under-five deaths in these annual hospitalisations significantly decreased from 5, 1% in 2009 to 1, 8% in 2018 (*p* < 0.001). The trend of this decrease is illustrated in Fig. 1.

**Fig. 2:**
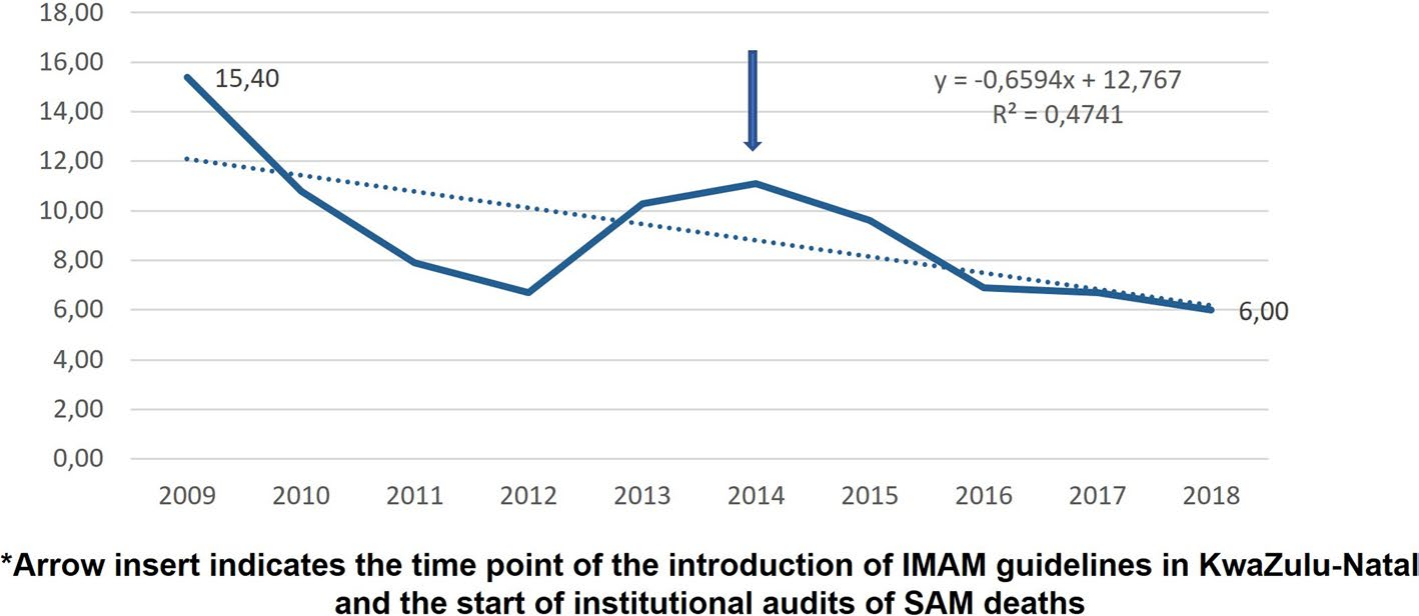
Changes in the proportion of SAM admissions as a percentage of all under-five admissions

Of all hospitalised children, 2328 (9, 1%) were classified as having severe acute malnutrition (SAM). The change in the proportion of SAM admissions as a percentage of all under-five admissions also showed a decrease over this period. Figure 2 illustrates this decrease.

**Fig. 3:**
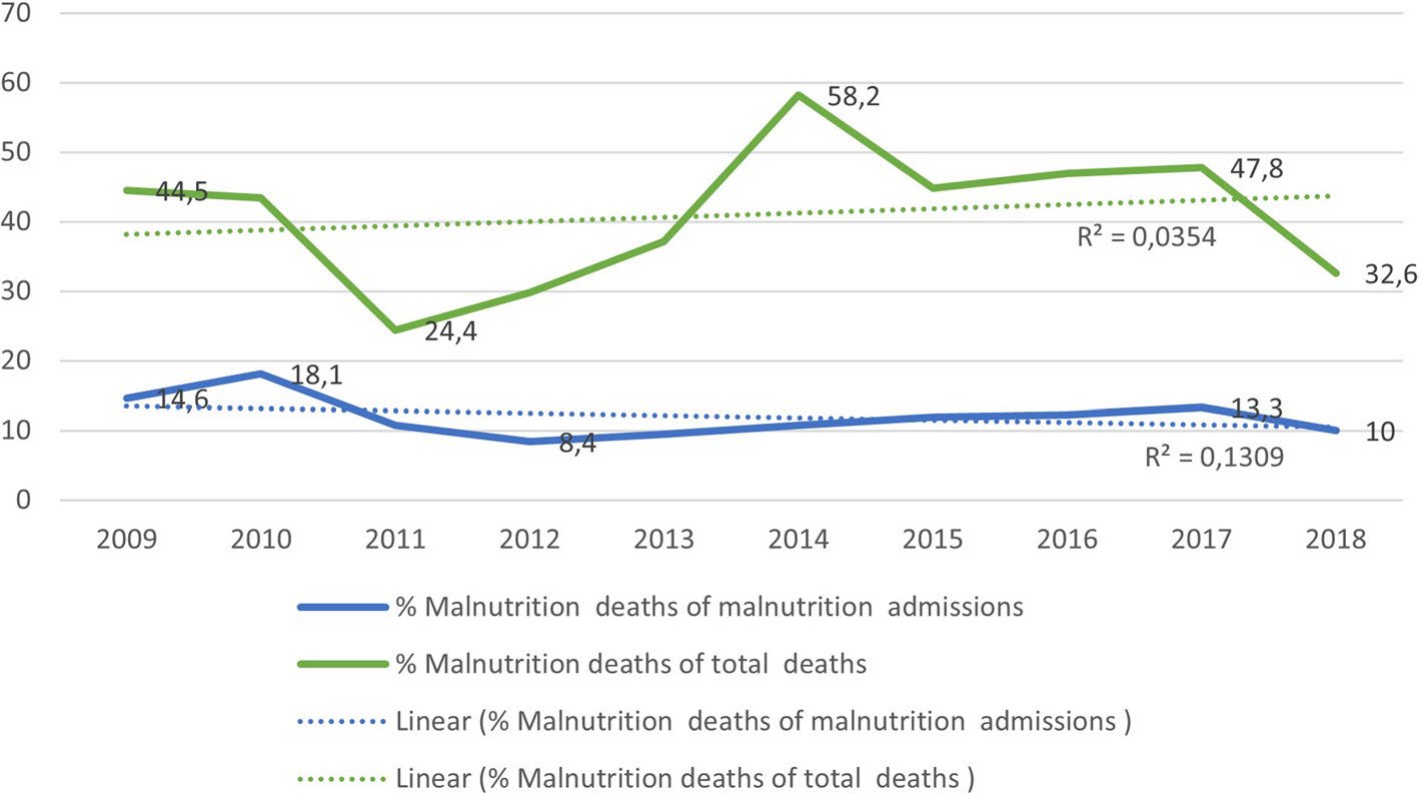
Trend analysis of the proportion of SAM under-five deaths as a percentage of all under-five deaths and as a percentage of SAM admissions

There were 285 SAM deaths during the study period. This constituted 12, 2% of all 2328 under-five SAM admissions and 40, 8% of all under-five deaths. From 2009 to 2018, there was no significant change in the proportion of SAM deaths, either as a percentage of SAM admissions (case fatality rate) or as a percentage of under-five deaths. For ten years, the mean case fatality rate was 12, 2% (range 8, 4% to 18, 1%), placing it well above the WHO target of < 5%. Figure 3 illustrates these trends.

Upon analysis of SAM deaths, *n* = 285, the mean age of children was 16,9 months, which was significantly different from the mean age of children classified in other nutritional categories who had died. This was significantly different from the ages of children who died who were classified as nutritionally normal. The gender in both the SAM (*n* = 285) and NAM (*n* = 301) categories was also significantly different. More boys had died in the SAM category (62, 7%) compared to NAM (49, 5%).

Table 1 indicates the relationship between age and gender in the death of children in various WHO nutritional categories.

**Table 1:** Demographic characteristics (age and gender) of the children classified in various nutritional categories

WHO	MAM (*N* = 79)	NAM (*N* = 301)	Obese (*N* = 19)	SAM (*N* = 285)	p-value	Overall (*N* = 684)
**Age (months)**					*p* < 0.001*	
Median (Q1-Q3)	5.00 (3.00–8.00)	7.00 (3.00–19.0)	17.0 (8.50–23.0)	10.0 (5.00–18.0)		8.00 (4.00–18.0)
Min–Max	1.00–46.0	1.00–59.0	1.00–36.0	1.00–58.0		1.00–59.0
**Gender**					*p* = 0.006	
Male	49 (62.0%)	147 (49.5%)	13 (68.4%)	178 (62.7%)		387 (57.0%)
Female	30 (38.0%)	150 (50.5%)	6 (31.6%)	106 (37.3%)		292 (43.0%)

^*^ Kruskal–Wallis test

Most under-five deaths, 60.7% (*n* = 415), occurred in HIV-exposed children. Of all the deaths, 49, 4% (338) were HIV negative, and 36, 8% (252) were HIV positive. In cases where the HIV status was known, most SAM deaths comprised HIV-positive children (46.7%), while the majority of deaths in children who were not classified as SAM were HIV-negative. Table 2 indicates the relationships between HIV exposure and HIV infection status in various WHO nutritional categories.

**Table 2:** The relationship between HIV exposure and HIV infection status in various WHO nutritional categories

**WHO**		**MAM (*N* = 79)**	**Normal (*N* = 301)**	**Obese (*N* = 19)**	**SAM (*N* = 285)**	**p-value**	**Overall (*N* = 684)**
**Exposed**						*p* = 0.373	
No		24 (30.4%)	123 (40.9%)	7 (36.8%)	115 (40.4%)		269 (39.3%)
Yes		55 (69.6%)	178 (59.1%)	12 (63.2%)	170 (59.6%)		415 (60.7%)
**HIV status**						*p* < 0.001	
Negative		40 (50.6%)	172 (57.1%)	16 (84.2%)	110 (38.6%)		338 (49.4%)
Positive		30 (38.0%)	87 (28.9%)	2 (10.5%)	133 (46.7%)		252 (36.8%)
Unknown HIV		9 (11.4%)	42 (14.0%)	1 (5.3%)	42 (14.7%)		94 (13.7%)

**Fig. 4:**
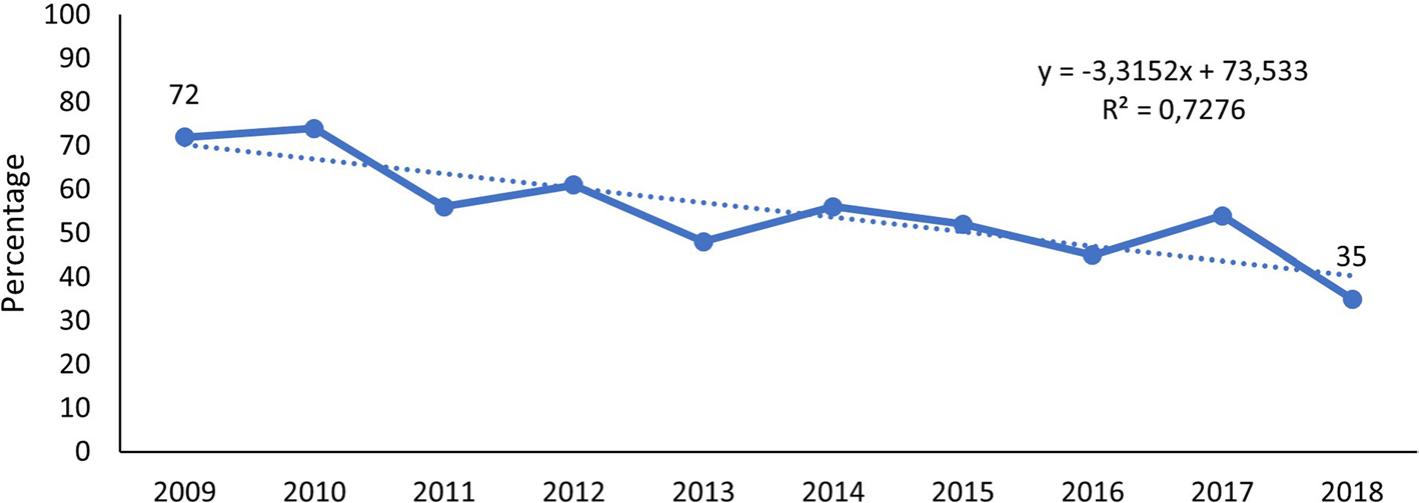
Changes in HIV exposure percentage in SAM deaths

There was a significant decrease in the percentage of HIV-exposed SAM deaths from 2009 (72%) to 2018 (35%). Figure 4 illustrates this change.

**Fig. 5:**
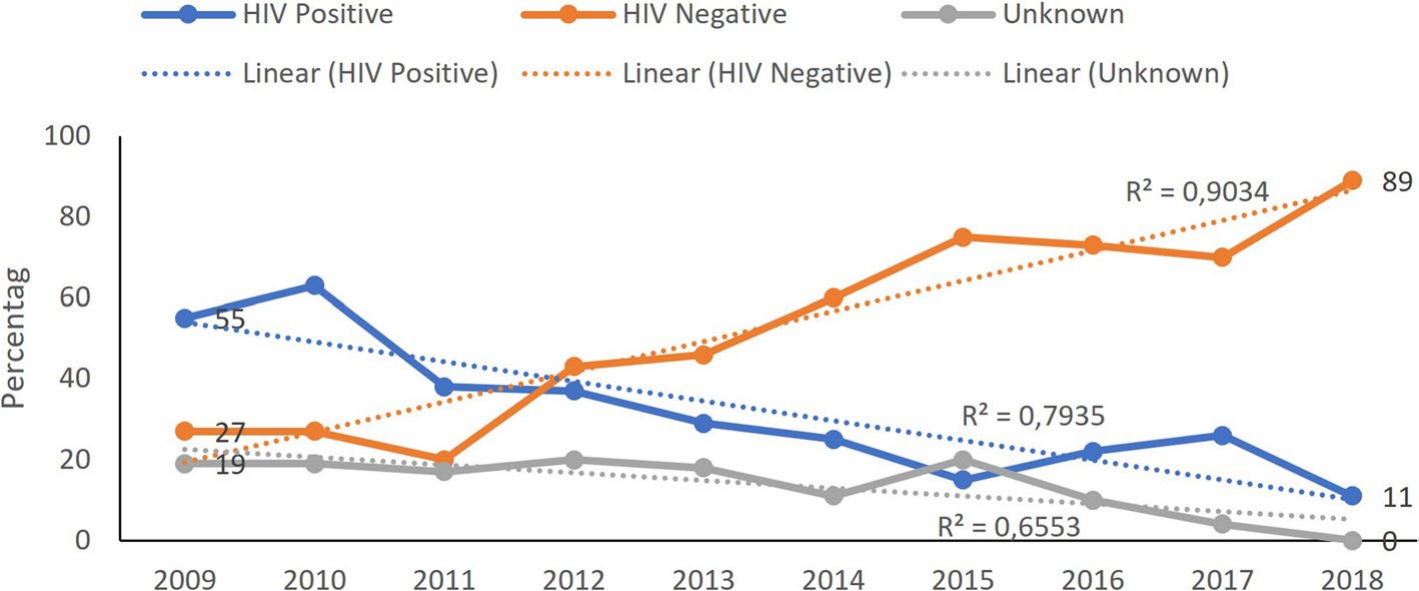
Changes in HIV-exposed and infected children with SAM who died. (2009–2018)

**Fig. 6:**
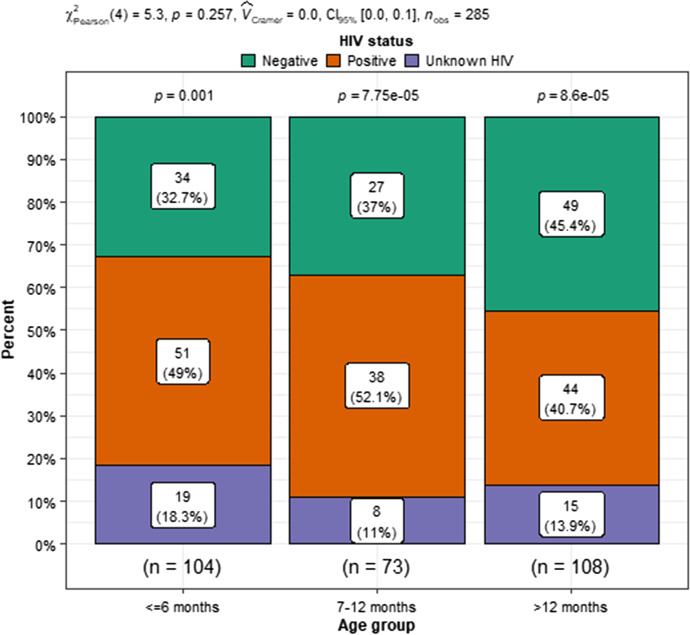
Comparing the relationship of age and HIV status with SAM nutritional status

The proportion of HIV-positive, HIV-negative, and HIV unknown SAM deaths changed over the study period. This change was statistically significant. Figure 5 illustrates these trends. In 2009, most (70, 2%) SAM deaths were HIV infected, compared with only 6, 7% of SAM deaths in 2018. There was also a significant drop in HIV unknown results from 19,8% of all SAM deaths not having an HIV result documented in 2009 to no SAM deaths having no HIV result reported in 2018. In analysing the severely acutely malnourished children who died in relation to both age and HIV status, there was a significant association between being below six months and HIV positive. Figure 6 illustrates these relationships.

### Cause of death

**Fig. 7:**
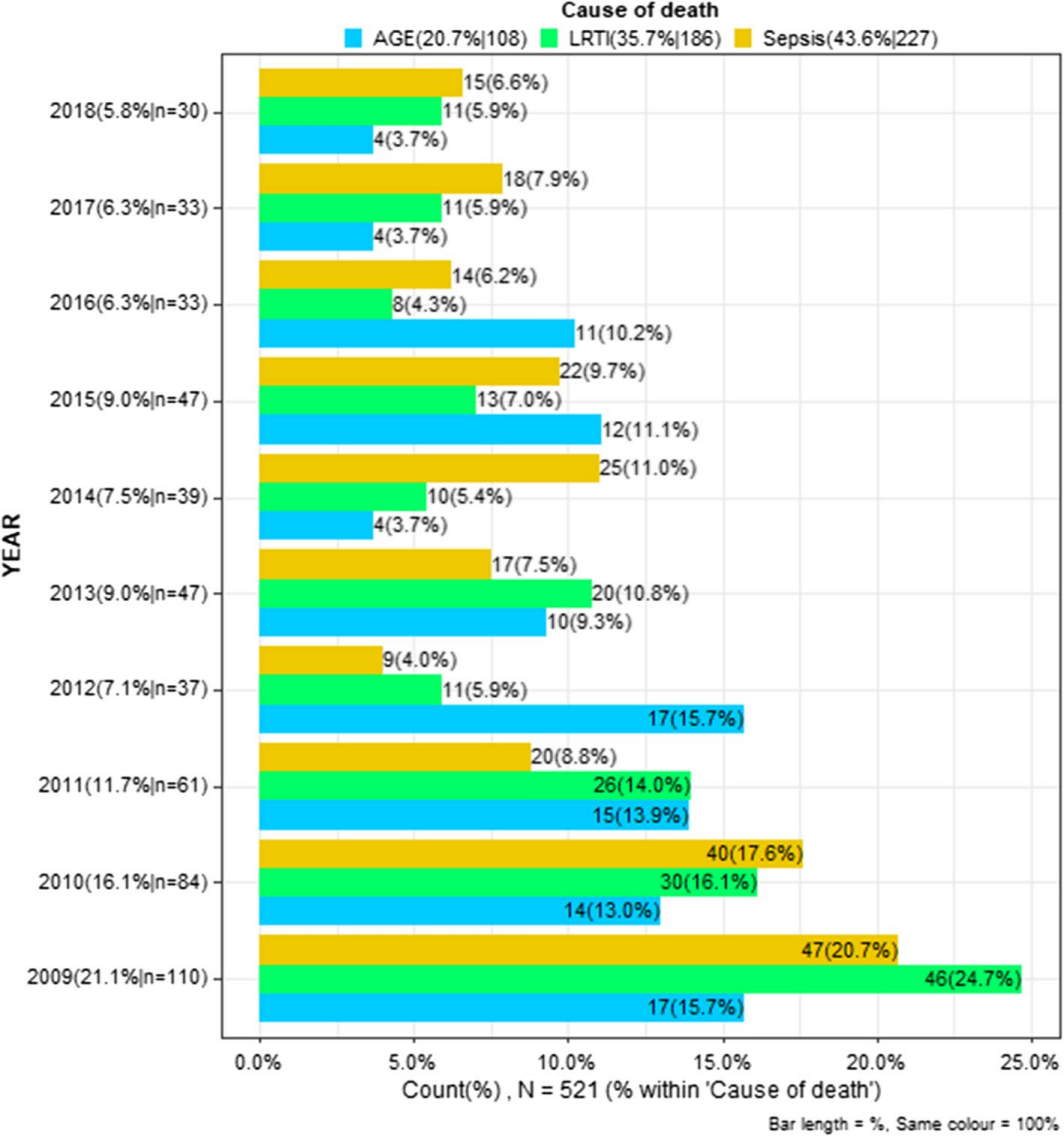
Change in the top three causes of death from 2009 until 2018 in all children who died

The top three primary causes of death were septicaemia (33%, *n* = 223) and lower respiratory tract infections (27%, *n* = 182), followed by acute gastroenteritis (15%, 7%, *n* = 106). Significantly more children died from septicaemia as the primary cause of death 41, 6%, (*n* = 117) in SAM children who died compared with 25, 3% (*n* = 75) in children classified as NAM. Table 3 indicates the proportion of primary causes of death in each nutritional category. There were no significant changes in the proportions of the three most common causes of death in either the SAM or NAM groups over the study period. Figure 7 illustrates changes in these proportions over the ten years.

**Table 3:** Causes of death from 2009 until 2018 in all children who died

	**WHO nutritional classification categories**					
**Primary cause of death**	**MAM (*N* = 79)**	**NAM (*N* = 301)**	**Obese (*N* = 19)**	**SAM (*N* = 285)**	**Overall (*N* = 684)**	
Acute Gastroenteritis (AGE)	9 (11.5%)	47 (15.8%)	7 (36.8%)	43 (15.3%)	106 (15.7%)	*p*-value = 0.044
Lower Respiratory Tract Infections (LRTI)	24 (30.8%)	94 (31.6%)	4 (21.1%)	60 (21.4%)	182 (27.0%)	
Confirmed /Suspected Septicaemia (Sepsis)	28 (35.9%)	75 (25.3%)	3 (15.8%)	117 (41.6%)	223 (33.0%)	
Meningitis	5 (6.4%)	13 (4.4%)	0 (0.0%)	12 (4.3%)	30 (4.4%)	
Cardiac failure	4 (5.1%)	23 (7.7%)	3 (15.8%)	15 (5.3%)	45 (6.7%)	
Neurological causes	4 (5.1%)	22 (7.4%)	1 (5.3%)	13 (4.6%)	40 (5.9%)	
Liver Failure /disease	2 (2.6%)	11 (3.7%)	1 (5.3%)	13 (4.6%)	27 (4.0%)	
Renal Failure /disease	0 (0.0%)	3 (1.0%)	0 (0.0%)	3 (1.1%)	6 (0.9%)	
Other causes	2 (2.6%)	9 (3.0%)	0 (0.0%)	5 (1.8%)	16 (2.4%)

## Discussion

From 2009 to 2018, significant changes in the characteristics of hospitalised children with severe acute malnutrition who died in KEH were identified in this study.

The decrease in under-five in-hospital mortality at KEH over the study period was significant and consistent with national and international trends [2, 3]. In this study, there was a significant decrease in the proportion of SAM hospitalisations. This mirrors trends in other Sub-Saharan countries and may suggest that programmes to prevent HIV and/or malnutrition at the primary care level could possibly be making an impact [6]. However, previous reports evaluating community levels of undernutrition have documented persistent levels of poor nutrition [20, 27]. These studies reflected the impact of various population-based nutritional programmes in SA before the full-scale implementation of HIV prevention/treatment programmes and hospital-level nutrition programmes. The decrease in SAM hospitalisations, specifically from 2014, identified in this study, coincides with the introduction and implementation of the IMAM guidelines that focus on in-hospital audits and specific protocols for managing children with SAM. However, this postulate needs to be treated with caution, as referral hospitals often admit SAM children with underlying pathologies, which may not reflect all hospitalised SAM patients. The independent and interactive effects of HIV infection, especially on survival in these children, need further exploration [23].

This study demonstrates that the proportion of HIV-infected children with SAM who died in KEH significantly changed as the ART prevention and treatment programmes reached both scale and intensity. This change was particularly noted in the under six-month age group, where a large component of under-five mortality occurs. The interaction of HIV infection, critical illness and malnutrition have been documented to be the greatest risk for survival [6, 23]. This study corroborates these findings and describes a longitudinal trend that associates early HIV prevention, identification and treatment programmes with a decrease in HIV-infected SAM deaths.

The concerning aspect that this study identified is the lack of a sustained decrease in the percentage of children with SAM who demised both as a proportion of all SAM hospitalisations or as a proportion of all under-five deaths annually. The high case fatality rate for children with SAM in this cohort is similar to studies from other developing countries. However, the lack of change occurs despite improvements in HIV-related morbidity and mortality [18, 19]. Inpatient management for all malnourished children focuses on strategies that include careful use of ready-to-use feeds and immediate acute care standardised protocols [19]. In this study, non-HIV-related SAM deaths were identified as being of specific concern and need further analysis and investigation.

Based on aetiology, SAM can be either illness-related (secondary to one or more diseases/injury), non-illness-related (caused primarily by food insecurity) or both [28, 29]. With HIV-positive and HIV-negative SAM children often exposed to similar socioeconomic conditions, it seems highly plausible that other underlying medical conditions may also contribute to mortality in HIV-negative children. With most deaths in children with SAM now occurring in HIV-negative children in KEH, a greater focus on identifying any underlying pathologies may be needed. Tuberculosis, which has often been suspected but rarely proven to be diagnosed in malnourished children, is an underlying pathology that may be overlooked [30]. Other underlying pathologies noted in SAM admissions and deaths include neurological conditions, malabsorption syndromes, cardiac abnormalities, genetic defects, and urinary tract infections with septicaemia [29, 31, 33].

Inpatient nutrition protocols for SAM, including the IMAM guidelines, have primarily focused on standard protocols for managing both ‘primary illness-related’ SAM and ‘secondary illness-related’ SAM similarly. [17, 27]. However, further research is urgently required for many SAM children admitted to referral hospitals that fail to improve on standard WHO protocols. We postulate that this research could possibly influence changes to existing WHO protocols in managing SAM. In addition, these protocols need to include an ability to individualise management principles, especially to align to possible underlying pathologies contributing to malnutrition.

### Limitations

Retrospective data were used to analyse this study, where no verification or standardisation of anthropometric measurements was recorded. Data on mid-upper-arm circumferences (MUAC) were not used, as this was inconsistent in documentation over the years, and standalone MUAC is not ideal [34]. Stunting information was not analysed, as height measurements could not be standardised or verified over this period. The study did not include verified birth weight nor breastfeeding data, especially for the first six months, and the data are from one province. HIV information was recorded in source case notes or mortality summaries and was not verified from laboratory sources over the entire study period. An interrupted time series analysis was not performed on the data and may of added increased insight**.**

## Conclusion

The significant decreases noted between 2009 and 2018 in the mortality of hospitalised children in KEH can be attributed to effective HIV treatment and prevention programmes. While some decreases are noted in the rates of hospitalisation of children with SAM, the cause for concern is the lack of significant change in the case fatality rate of these children despite multiple nutritional programmatic interventions. Increasingly more SAM children who die are HIV negative, with possible underlying medical pathologies being overlooked. Standardised protocols currently in place do not differentiate between illness-related and non-illness-related malnutrition, even in referral hospitals. Modification of national malnutrition guidelines for this at-risk population may need to be considered in referral hospitals in South Africa.

## Data Availability

The data supporting this study’s findings are available from the corresponding author upon reasonable request.
